# Hereditary breast cancer associated with Cowden syndrome-related PTEN mutation with Lhermitte-Duclos disease

**DOI:** 10.1186/s40792-017-0355-6

**Published:** 2017-07-24

**Authors:** Fuyo Kimura, Ai Ueda, Eiichi Sato, Jiro Akimoto, Hiroshi Kaise, Kimito Yamada, Mari Hosonaga, Yuko Kawai, Saeko Teraoka, Miki Okazaki, Takashi Ishikawa

**Affiliations:** 1 Department of Breast Oncology and Surgery, Tokyo Medical University, 6-7-1 Nishishinjuku, Shinjuku-ku, Tokyo, 160-0023 Japan; 2 Department of Anatomic Pathology, Tokyo Medical University, 6-7-1 Nishishinjuku, Shinjuku-ku, Tokyo, 160-0023 Japan; 3 Department of Neurosurgery, Tokyo Medical University, 6-7-1 Nishishinjuku, Shinjuku-ku, Tokyo, 160-0023 Japan; 4 Department of Breast Surgery, The Second Kawasaki Saiwai Clinic, 39-1 Tocho, Saiwai-ku, Kawasaki, Kanagawa 212-0021 Japan

**Keywords:** Lhermitte-Duclos disease, Cowden syndrome, Breast cancer, Hereditary breast cancer, *PTEN* mutation

## Abstract

**Background:**

Cowden syndrome is characterized by multiple hamartomas in various tissues, including the skin, brain, breast, thyroid, mucous membrane, and gastrointestinal tract, and is reported to increase the risk of malignant disease.

**Case presentation:**

We describe the case of a 52-year-old woman in whom a tumor was diagnosed in the left cerebellar hemisphere and treated by surgical resection. Phosphatase and tensin homolog (*PTEN*) mutation in exon 8 insertion was found in the brain tumor tissue and leukocytes. This finding supported the diagnosis of Cowden syndrome. She consequently developed endometrial cancer and underwent abdominal total hysterectomy with bilateral salpingo-oophorectomy. Four years later, hormone receptor-positive breast cancer was found in the right breast, and breast-conserving surgery with radiation therapy and sentinel lymph node biopsy was performed.

**Conclusions:**

Herein, we describe a patient who was diagnosed as having familial breast cancer associated with *PTEN* mutation-related Cowden syndrome. We also reviewed reports of this syndrome in the literature for disease appraisal.

## Background

Estimates show that up to 15% of breast cancer patients have one or more first-degree relatives with breast cancer [1]. Inherited breast cancer is caused by penetrant susceptibility genes and most often involves germ line mutations of the *BRCA1* and *BRCA2* genes in about 15% of familial breast cancer patients. Tumor protein 53 (*TP53*), cadherin 1 (*CDH1*), liver kinase B1 (*LKB1*), and phosphatase and tensin homolog (*PTEN*) are rarely associated with the development of breast cancer, which occurs in only about 3% of patients with familial breast cancer. Breast cancer in approximately half of women with a familial history may also result from unexplained genes [2].

Cowden syndrome (CS) is an autosomal dominant inherited cancer syndrome associated with germ line mutations in *PTEN*, a tumor suppressor gene. CS is characterized by multiple hamartomas and developing breast, thyroid, and endometrial malignancies. Dysplastic cerebellar gangliocytoma called Lhermitte-Duclos disease (LDD) is also associated with CS [3, 4]. The cumulative lifetime risks of any cancer diagnosis is 89% for CS patients, and the morbidities are 85% in breast cancer, 32% in LDD, 21% in thyroid cancer, 19% in endometrial cancer, 15% in renal cancer, 16% in colon and rectum cancers, and 15% in kidney cancer [4, 5]. Herein, we report a case of *PTEN*-mutated hereditary breast cancer with LDD.

## Case presentation

**Fig. 1:**
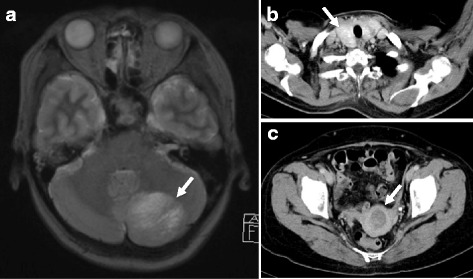
MRI and CT at the age of 50 years. **a** MRI showed alternating isointense and hyperintense bands in the left cerebellar hemisphere (*arrow*). **b** Whole-body CT screening detected an adenomatous goiter (*arrow*). **c** CT also demonstrated uterine cancer (*arrow*)

**Fig. 2:**
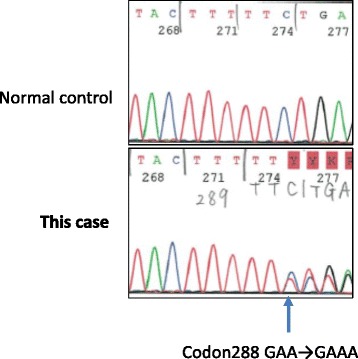
Analysis of *PTEN* gene mutations. Genomic DNAs for germ line mutation analyses were obtained from the buffy coat and resected tumor. Polymerase chain reaction products were sequenced directly using an automated DNA-sequencing system (Model 3130; Applied Biosystems, Foster City, CA). *PTEN* sequencing demonstrated that a germ line mutation c. 288 insertion A was found in exon 8, which caused a frameshift mutation (p.V290fs*8). A reverse primer was used in this analysis

The patient was a 52-year-old woman with a prior history of chronic thyroiditis. Her sister was diagnosed as having congenital hydrocephalus and her mother as having heart valve disease. Her father died of myocardial infarction. The patient had a fall and sustained a head injury. Brain T2-weighted magnetic resonance imaging (MRI) revealed alternating isointense and hyperintense bands in the left cerebellar hemisphere (Fig. 1a). Surgical resection of the tumor in the cerebellar hemisphere was performed, and the histopathological diagnosis was dysplastic gangliocytoma. The patient was subsequently referred for genetic counseling. Genetic testing of her leukocytes and resected tumor revealed mutation in the exon 8 insertion of the *PTEN* gene (Fig. 2), leading to a diagnosis of CS associated with LDD.

**Fig. 3:**
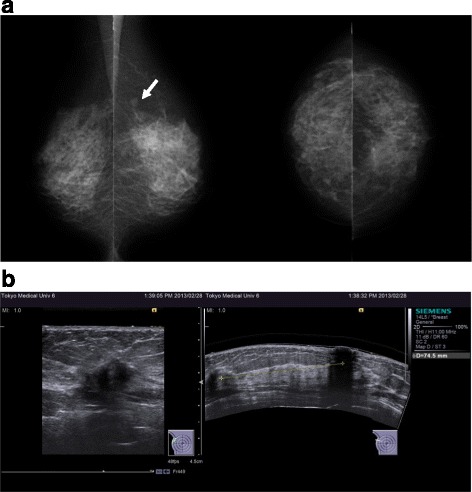
MMG and US examinations at the age of 55 years. **a** A smooth mass was detected in the left breast by MMG. **b** An irregular indistinct mass (12 × 11 × 10 mm) was found in the right breast by US

**Fig. 4:**
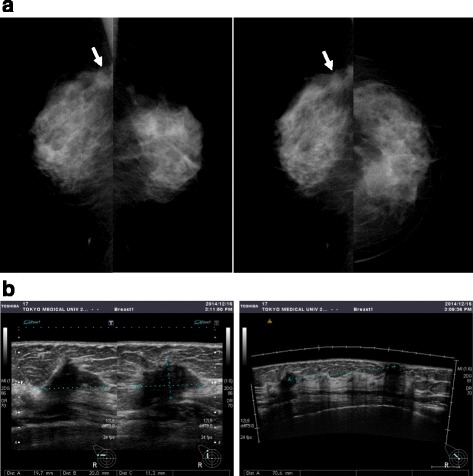
MMG and US at the age of 57 years. **a** A spiculated mass was observed in the right breast. **b** The size of the irregular indistinct tumor in the right breast increased (20 × 20 × 11 mm) as shown by US

After genetic diagnosis, computed tomography (CT) whole-body screening revealed an adenomatous goiter (Fig. 1b) and uterine cancer (Fig. 1c). Thus, abdominal total hysterectomy with bilateral salpingo-oophorectomy was performed. The histopathological finding was endometrioid adenocarcinoma with no lymph node metastasis. At the age of 55 years, the follow-up CT for uterine cancer incidentally revealed a tumor in the right breast. Mammography (MMG) showed a smooth mass in the left breast (Fig. 3a). An irregular indistinct mass (12 × 11 × 10 mm) was detected in the right breast by ultrasonography (US) (Fig. 3b). However, she did not visit our hospital for the required additional examination. Two years later, a breast mass was detected in the breast cancer screening. A spiculated mass was observed in the right breast on MMG (Fig. 4a), and the tumor increased in size (20 × 20 × 11 mm) as shown by US (Fig. 4b). Core needle biopsy revealed invasive ductal carcinoma: estrogen and progesterone receptor-positive, Her2 2+, and Ki-67 20%. The patient underwent breast-conserving surgery and sentinel lymph node biopsy. Histopathological examination confirmed an invasive micropapillary carcinoma measuring 2.5 × 2.1 × 1.3 cm.

**Fig. 5:**
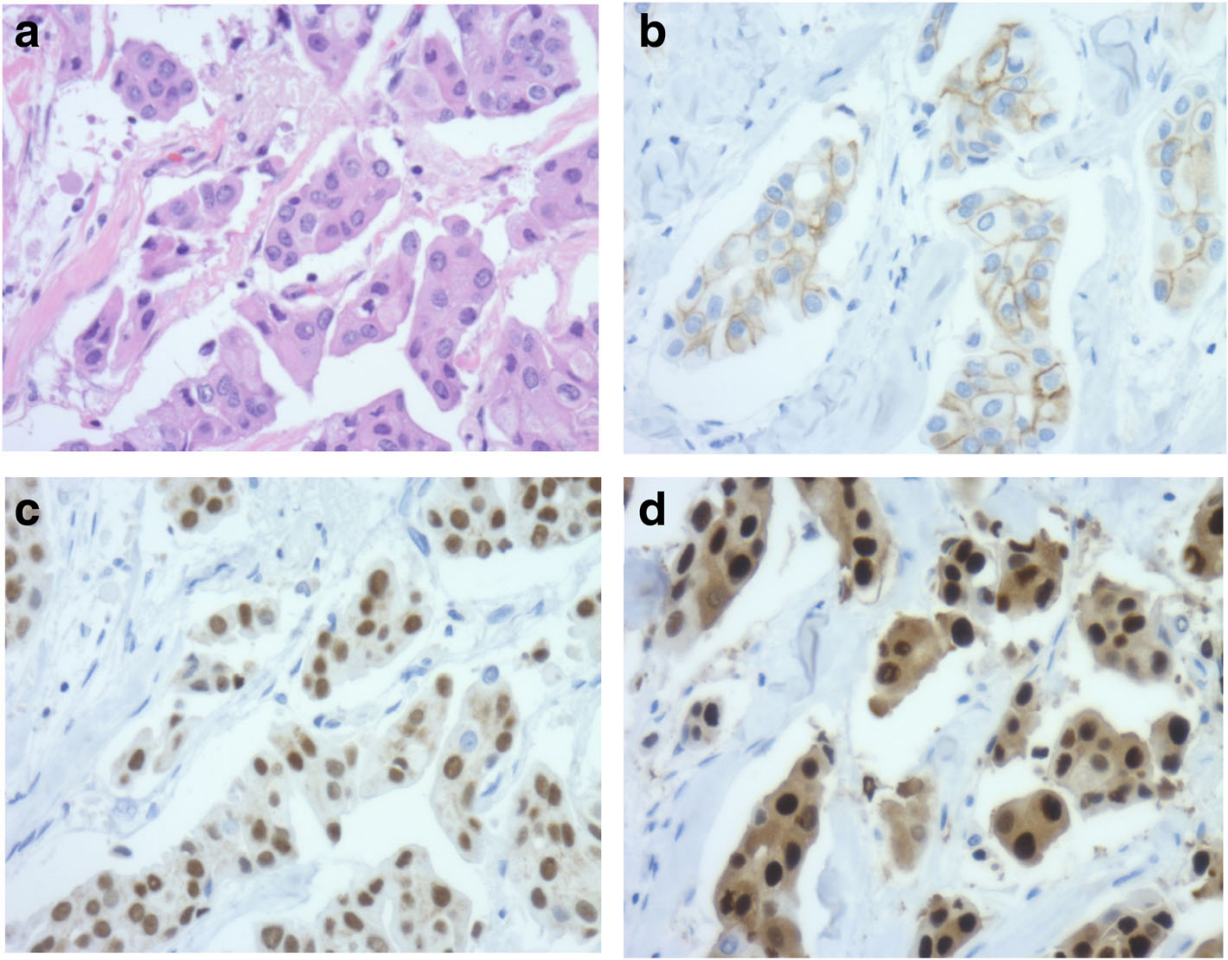
Histopathological features of the tumor in this case. **a** Invasive micropapillary carcinoma (H&E staining, ×200). **b** Immunohistochemistry showed Her2 receptor 2+ (×200). **c** Estrogen receptor-positive staining of 100% (×200). **d** Progesterone receptor-positive staining of 100% (×200)

Immunohistochemistry revealed estrogen receptor-positive and progesterone receptor-positive staining, Her2 2+, and Ki-67-positive staining of 10% (Fig. 5). The lymph nodes were not involved. She was given 50 Gy radiation and hormone therapy as adjuvant therapy. Any sign of recurrent disease has not been found in the 3 years since the last surgery for her breast cancer.

### Discussion

Previous studies have shown that approximately 20 to 34% of patients with CS carry germ line mutations of *PTEN*, a gene located on chromosome 10q23 [6–8]. These include 19–29% missense mutations, 19–33% nonsense mutations, 20% insertions, 2–14% small deletions, 10–11% splice site mutations, 1–29% frameshift mutations, 3% large deletions, and 3% promoter mutations [6, 7, 9]. In the present case, adenine was inserted in the codon of exon 8, resulting in the rearrangement of the amino acid sequence from valine to serine (Fig. 2). There were 27 reported cases of CS with deleterious mutation in Japan from 1984 to 2017 (May) according to *Igaku Chuo Zasshi* which is updated by the Japan Medical Abstracts Society.

PTEN participates in regulating the phosphoinositide 3 kinase (PI3K)-AKT and mTOR signaling pathways for negative regulation, controls cell proliferation and cell cycle progression, and promotes apoptosis. Thus, a loss of PTEN function correlates with the development of various human cancers [10]. It has been reported that *PTEN* is a gene in which mutation is most frequently found in primary breast cancers [11]. It remains controversial how the abnormality of this gene function may affect the prognosis of breast cancer patients. Previous reports of cases with CS revealed that most breast cancers were hormone receptor-positive including the present case (Table 1) [12–25]. Based on the history of the present case representing a slow-growing tumor, the clinical course of this case may be favorable. CS has a high risk of transforming into malignant tumors; thus, cancer surveillance is an important management for other organs [26]. The National Comprehensive Cancer Network (NCCN) recommendation is to undergo thyroid ultrasound, colonoscopy, renal ultrasound, annual dermatologic examination, and annual endometrial biopsies. After genetic diagnosis, we performed screening for breast, thyroid, and endometrial malignancies. Endometrial adenocarcinoma was found. Usually, the recommended treatment for hereditary breast cancer associated with *BRCA1* mutation is mastectomy because it is a more aggressive cancer with a poor prognosis. However, as *PTEN*-related breast cancer tends to grow slowly, patients are not always required to undergo mastectomy. We therefore performed breast-conserving surgery in the present case.

**Table 1:** Expression of hormone receptor in Cowden disease after 2000

Case	Age at surgery (Sex)	ER	PR	Her2	Pathology	Stage	Bilateral subtype	PTEN mutation
Kanayama Y, et al. (2011) [12]	61 (F)	+	Unknown	−	Invasive ductal carcinoma	T4cN2aM0	ER-PR-Her2-	Exon 5 exon 7 mutation
Morse CB, et al. (2015) [13]	40 (F)	n/a	n/a	n/a	ADH			Missense mutation
Kalin A (2013) [14]	37 (F)	+	+	−	Invasive ductal carcinoma	Stage IIb		Mutation
Peiró G, et al. (2010) [15]	44 (F)	+	+	−	Invasive ductal carcinoma	T2N0M0	ER+PR+Her2-	Exon 8 splice-acceptor site mutation
Erickson J, et al. (2010) [16]	39 (F)	+	+	Unknown	Invasive ductal carcinoma	Stage II		n/a
Winter H, et al. (2012) 17	35 (F)	+	+	−	Invasive ductal carcinoma	T3N1M1		Frameshift mutation
Seo M, et al. (2014) [18]	22 (F)	n/a	n/a	n/a	DCIS			Frameshift mutation
Sabaté JM, et al. (2006) [19]	42 (F)	−	−	+	Invasive ductal carcinoma	T2N0M0		Mutation
Sabaté JM, et al. (2006) [19]	38 (F)	−	−	+	Invasive ductal carcinoma	T1N0M0		Mutation
Ball S, et al. (2001) [20]	38 (F)	+	Unknown	Unknown	Invasive ductal carcinoma	T2N0M0	DCIS	n/a
Walsh S, et al. (2011) [21]	34 (F)	+	Unknown	−	Invasive ductal carcinoma	T2N0M0		Exon 7 deletion and insertion
Fackenthal JD, et al. (2001) [22]	41 (F)	+	Unknown	Unknown	Invasive ductal carcinoma			Exon 7 splicing mutation
Baù MG, et al. (2004) [23]	50 (F)	+	Unknown	Unknown	Invasive ductal carcinoma	T1bN1aM0		Mutation
Nakamura T, et al. (2012) [24]	38 (F)	+	+	−	Invasive ductal carcinoma	T1N0M0		Exon 5 missense mutation
Kikuchi S, et al. (2014) [25]	40 (F)	+	+	−	Invasive ductal carcinoma	T1cN0M0		Exon 7 nonsense mutation
Kimura F, et al. (2017) [this case]	55 (F)	+	+	−	Invasive ductal carcinoma	T2N0M0		Exon 8 insertion

*F* female, *M* male, *ADH* atypical ductal hyperplasia, *DCIS* ductal carcinoma in situ

The American Cancer Society and NCCN guidelines recommend MRI in addition to MMG for high-risk patients including those with *PTEN* hamartoma tumor syndrome [27, 28]. It may be necessary to apply screening of breast MRI to patients with a high risk for high-grade hereditary breast cancers such as *BRCA1* mutation carriers. However, screening breast MRI may not be practical to apply to all hereditary breast cancer cases because of its relatively low specificity and the high cost of enhanced breast MRI. It is also difficult to perform pathological examination if the lesion is detected only by MRI.

However, the usual screening management for breast cancer may be insufficient for patients with CS, because the mean age at diagnosis of breast cancer is reported to be about 40 years old in women with CS [29], which is younger than in the usual population. Thus, it is necessary to consider starting MMG screening at an earlier age and performing it more frequently such as once a year. Ultrasound could be another option, which may be useful for younger women and women with dense breasts [30].

## Conclusions

In conclusion, we report the case of a rare familial breast cancer syndrome of CS. Unlike other familial breast cancers such as those with *BRCA1* mutation, breast cancers with CS are generally hormone receptor-positive and may have a favorable clinical course.

## Abbreviations

CS: Cowden syndrome
CT: Computed tomography
LDD: Lhermitte-Duclos disease
MMG: Mammography
MRI: Magnetic resonance imaging
PTEN: Phosphatase and tensin homolog
US: Ultrasonography
